# Impact of flooding events on waterborne and vector-borne infections: a systematic review

**DOI:** 10.1186/s12879-026-13442-z

**Published:** 2026-04-28

**Authors:** Athitaya Luangnara, Saikhuan Towachiraporn, Nutchar Wiwatkunupakarn, Harit Thongwitokomarn

**Affiliations:** 1 Department of Internal Medicine, Faculty of Medicine, Chiang Mai University, Chiang Mai, Thailand; 2 Department of Family Medicine, Faculty of Medicine, Chiang Mai University, Chiang Mai, Thailand; 3 Division of Infectious Diseases and Tropical Medicine, Department of Internal Medicine, Faculty of Medicine, Chiang Mai University, Chiang Mai, Thailand

**Keywords:** Flooding, Vector-borne infections, Waterborne infections

## Abstract

**Background:**

Flooding from natural disasters has become increasingly common worldwide, increasing the risk of infectious disease transmission, particularly waterborne and vector-borne infections. Although many studies report outbreaks following floods, the evidence remains fragmented across regions and pathogens. A comprehensive review is needed to clarify these associations and inform effective prevention and response strategies.

**Methods:**

A systematic search of PubMed, Embase, and the Cochrane Library was conducted between November 2024 and January 2025, identifying studies published from January 1, 2014, to December 31, 2024. Eligible studies assessed the association between flooding and waterborne or vector-borne infections and their health outcomes or evaluated related prevention and response interventions. Data were synthesized narratively due to study heterogeneity.

**Results:**

Of the 1,785 records identified, 71 studies met the inclusion criteria. Flooding was consistently associated with increased incidence of waterborne infections, including leptospirosis, cholera, bacillary dysentery, and hepatitis A/E, as well as vector-borne infections such as dengue and malaria. Outbreaks varied by region, flood characteristics, and the strength of existing public health infrastructure. The lag period between the onset of flooding and disease emergence ranged from days to weeks. Timely interventions such as chemoprophylaxis, vaccination, water, sanitation, and hygiene (WASH) measures, and vector control may help mitigate outbreaks; however, most included studies were observational in design, and the overall certainty of evidence was low to moderate. Data on health system impacts, including hospitalization rates, intensive care unit burden, and mortality stratified by level of care, remain limited.

**Conclusion:**

Flooding was most frequently associated with increased incidence of cholera, bacillary dysentery, leptospirosis, dengue, and malaria, each with distinct lag periods critical for outbreak prediction. Reported lag periods ranged from days to weeks. Limited observational evidence suggests that doxycycline chemoprophylaxis for leptospirosis, oral cholera vaccination, and early vector control may be useful in selected post-flood settings. These findings support strengthened post-flood surveillance and context-specific preparedness measures, while highlighting the need for higher-quality comparative studies.

**Registration:**

PROSPERO CRD42025635687.

**Clinical trial numbe:**

Not applicable.

**Supplementary Information:**

The online version contains supplementary material available at 10.1186/s12879-026-13442-z.

## Background

In recent years, flooding from natural disasters has occurred globally, possibly due to population growth and climate change [1, 2]. From 1990 to 2022, 4,713 floods were recorded in 168 countries and territories, affecting more than 3.2 billion people and resulting in 218,353 deaths [3]. Beyond the immediate destruction of infrastructure and economic losses, flooding significantly increases the risk of infectious disease transmission, particularly waterborne and vector-borne infections [4]. Waterborne infections are caused by the ingestion of, or contact with, water contaminated with pathogenic microorganisms, and include diseases such as cholera, typhoid fever, hepatitis A, and leptospirosis [5]. Vector-borne infections are transmitted by living organisms, most commonly mosquitoes, that carry pathogens from one host to another, leading to diseases such as malaria, dengue, chikungunya, and Zika virus disease [6]. These outbreaks contribute substantially to post-disaster morbidity and mortality, posing significant public health concerns.

Although numerous studies have documented infectious disease outbreaks following flood events, the evidence remains fragmented across regions and disease types. To date, no comprehensive synthesis has examined both waterborne and vector-borne infections in relation to flooding worldwide between 2014 and 2024, nor evaluated their health system impacts, such as increased hospitalizations or ICU burden. A systematic synthesis of the existing literature is therefore needed to clarify the epidemiological patterns, contributing factors, and healthcare implications of flood-associated infections. This review aims to systematically synthesize existing evidence on the association between flooding and waterborne and vector-borne infections across different geographic and epidemiologic contexts. By consolidating fragmented data, it seeks to identify flood characteristics and lag periods, outbreak patterns and epidemiology, health system impact, and prevention and response measures thereby offering decisive information for public health preparedness and policy-making in flood-prone regions.

## Methods

This systematic review was conducted in accordance with a predefined protocol and reported following the Preferred Reporting Items for Systematic Reviews and Meta-Analyses (PRISMA) guideline [7]. The review protocol was registered in the International Prospective Register of Systematic Reviews (PROSPERO) (registration number: CRD42025635687). This research project was exempted from ethical review by the Research Ethics Committee of the Faculty of Medicine, Chiang Mai University (Study Code: MED-2567-0706).

### Definitions

For the purpose of this review, the following definitions were applied:

**Flooding** was defined as a temporary overflow of water onto land that is normally dry, including events caused by heavy rainfall, river overflow, storm surges, or flash floods [8].

**Waterborne infections** were defined as infectious diseases transmitted through the ingestion of or contact with water contaminated with pathogenic microorganisms (e.g., cholera, typhoid fever, leptospirosis, hepatitis A) [5].

**Vector-borne infections** were defined as diseases transmitted by vectors such as mosquitoes or flea that carry pathogens from one host to another (e.g., malaria, dengue, chikungunya) [6].

### Search strategy and inclusion criteria

A systematic search was conducted in PubMed, Embase, and the Cochrane Library between November 2024 and January 2025 to identify studies published from January 1, 2014, to December 31, 2024. The search strategy combined Medical Subject Headings (MeSH) and keywords related to “flooding,” “waterborne infections,” *and* “vector-borne infections” using Boolean operators. The full search strategies are available in Supplementary File 1. The inclusion criteria were (1) original research articles, including cohort, case-control, cross-sectional studies, and randomized controlled trials; (2) studies involving human populations affected by flooding in any region; and (3) studies that assessed the association between flooding and the incidence, prevalence, or outbreaks of waterborne or vector-borne infections in human populations, or examined the impact of flood-related waterborne or vector-borne infections on disease prevention efforts and healthcare service delivery during flooding. In contrast, the exclusion criteria were (1) narrative reviews, editorials, systematic reviews, meta-analyses, and non-human studies; (2) studies focused exclusively on mental health, non-communicable diseases, or injuries related to flooding; and (3) studies published in languages other than English or outside the publication period of 2014 to 2024.

### Data extraction

Two independent reviewers (AL and HT) screened the titles, abstracts, and full texts according to predefined inclusion and exclusion criteria. Data extraction was performed using a standardized, piloted form to ensure consistency. Extracted variables included study characteristics (authors, year of publication, country), study design, exposure details (cause of flooding), year of event, and reported lag time between flooding and disease onset. Additional data were collected on identified risk factors, effect modifiers, evaluated interventions, and outcomes of interest—such as incidence, prevalence, outbreak occurrence, and the impact of flood-related waterborne and vector-borne infections on healthcare service delivery. Discrepancies were resolved through discussion or adjudication by a third reviewer (NW). Reference management and screening were conducted using Rayyan (Qatar Computing Research Institute, Doha, Qatar).

### Risk of bias assessment

Risk of bias was assessed using appropriate tools based on study design. The NIH Quality Assessment Tool was applied to case-control and cross-sectional studies, while the JBI Critical Appraisal Checklist was used for case reports and case series. For non-randomized studies comparing the effects of two or more interventions, the ROBINS-I tool was employed. All assessments were conducted independently by two reviewers (AL and HT). Discrepancies were resolved through discussion or, when necessary, adjudication by a third reviewer (NW). The risk of bias of each study was presented in Supplementary File 1 (Tables S1-S3 and Figure S1). Risk-of-bias plots were generated using Robvis, an online visualization tool that creates publication-ready traffic-light and summary plots for bias assessments [9]. Given the use of different appraisal tools tailored to study design, a unified numerical scoring system was not applied. Instead, risk-of-bias assessments were summarized descriptively and considered qualitatively during narrative synthesis. Findings from studies rated as moderate or high risk of bias were interpreted with greater caution, and greater emphasis was placed on effect estimates from studies rated as low risk of bias when drawing overall conclusions.

### Synthesis method and data analysis

Given the substantial heterogeneity in study designs, exposure definitions (e.g., flood severity, rainfall thresholds, and timing), outcome measures (laboratory-confirmed cases, clinical diagnoses, hospitalization), effect metrics (RR, OR, IRR, incidence rates), and lag specifications, quantitative meta-analysis was deemed inappropriate, as pooling such diverse estimates could yield misleading summary effects. Therefore, a structured narrative synthesis was conducted. The synthesis framework involved grouping studies by disease category: (1) waterborne infections (including leptospirosis, infectious diarrhea, hepatitis A/E, and other pathogens) and (2) vector-borne infections (stratified into dengue, malaria, and other vector-borne infections). To reduce conceptual heterogeneity, we synthesized evidence in three domains: (1) epidemiologic associations between flooding and infection outcomes, (2) health system impact, and (3) prevention/response interventions. Within each pathogen group, we report these domains separately. Comprehensive summary tables (Tables 1, 2, 3, 4, 5, 6 and 7) were used to facilitate cross-study comparison. Quantitative findings were summarized descriptively using reported measures such as percentages, relative risks (RRs), odds ratios (ORs), or incidence rates, presented as originally reported by each study due to variation in effect metrics. Interpretation emphasized the direction, magnitude, and statistical precision of reported effect estimates, with explicit consideration of confidence intervals. All analyses were independently reviewed and approved by all authors.

**Table 1:** Summary of outbreak patternd and epidemiology on reported leptospirosis cases

Study (year)	Study Design	Flood Description (Cause/Year)	Location Country (city)	Lag Period (d/wk/mo)	Epidemiological Outcome	Impact on Health Service
**Studies Reporting Relative Effect Estimates**						
Suwanpakdee (2015) [10]	Observational	Monsoon/ 2010–2012	Thailand	N/A	IRR (per 100,000) = ↑ 4.03 (2010), ↑ 1.65 (2011), ↔ 0.66 (2012)	N/A
Ding (2019) [11]	Retrospective ecological	Unknown /2005–2012	China	50 d	↑ OR = 1.14	N/A
Togami (2018) [12]	Retrospective	Tropical depressions / Jan - Mar 2012	Fiji	2–6 wk	+ Mean weekly cases: 91.1 vs. 27.0 (flood vs. non-flood); ↑RR = 3.37	+ hospitalization and death (not quantify)
**Studies Reporting Absolute Outcome Measures**						
Wynwood (2014) [13]	Observational	Cyclone Yasi/ 2011	Australia	1–2 wk	Cases: 154 (12 mo)	N/A
Gao (2016) [14]	Observational	Unknown/2007	China	N/A	No reported cases	N/A
Mohd Radi (2018) [15]	Observational	Unknown/ Dec 2014	Malaysia	2–3 mo	+Cases: 753 vs. 375 (Pre Vs post-flood)	N/A
Marinova-Petkova (2019) [16]	Case series	Hurricanes Irma and Maria/ Sep 2017	USA	7–21 d	Cases: 3	N/A
Chadsuthi (2021) [17]	Retrospective	Seasonal monsoon floods and rainfal/ 2010–2016	Thailand	4–12 wk	+ Cases (not quantify)	N/A
Becirovic (2022) [18]	Retrospective	Cyclone Tamara/May 2014	Bosnia and Herzegovina	N/A	↑Cases: 80	N/A
Taunton (2022) [19]	Observational	Heavy rainfall /early 2021	Australia	N/A	↑ Cases: 43 vs. 27.8 (5-y mean) and 29.4 (10-y mean); + Crude notification rate(per 100,000): 39.0 vs. 1.6 (nationally)	69.8% hospitalization; 9.3% ICU admission; no fatalities
Yamamoto (2023) [20]	Case report	Typhoon/ Sep 2022	Japan	2 wk	Case: 1	N/A
Tsai (2020) [21]	Retrospective	Typhoon Morakot/2009	Taiwan	N/A	+ Inc (per 100,000): 0.9	N/A
Poulakida (2024) [22]	Case series	Storm Daniel /Sep 2023	Greece	1–3 wk	+Cases: 13	N/A
Ifejube (2024) [23]	Retrospective	Southwest monsoon/ 2018–2019	India	17 d	+ Inc (per 100,000): Alappuzha 361 (2018), 203 (2019) Pathanamthitta 3,122 (2018), 522 (2019) (14.77, global average)	N/A
Jones (2024) [24]	Retrospective	Hurricane Fiona/ Sep 2022	Puerto Rico	1–5 wk	+Inc (per 100,000): 17 vs. 5; mean weekly cases: ↑10.4 vs. 2.9 (flood vs. non-flood)	↓Hospitalization: 72% vs. 93% (flood vs. non-flood)

Abbreviations: ↑: Statistically significant increase, ↓: Statistically significant decrease ↔: No change, d: day(s), Dec: December, Inc: incidence, ICU: intensive care unit, IRR: incidence rate ratio, Jan: January, Mar: March, mo: month(s), N/A: not available, RR: risk ratio, Sep: September, wk: week(s)

**Table 2:** Summary of reported outbreak patternd and epidemiology on reported infectious diarrhea cases

Study (year)	Study Design	Flood Description (Cause/Year)	Location Country (city)	Disease(s)	Lag Period (d/wk/mo)	Epidemiological Outcome	Impact on Health Service
**Studies Reporting Relative Effect Estimates**							
Dalhat (2014) [25]	Retrospective	Unknown/ 2010	Nigeria	Cholera	N/A	+AR (per 100,000) = 47.8	CFR: 5.1%
Deng(2015) [26]	Case-crossover	7 Tropical cyclones /2005–2011	China	Bacillary dysentery	6 d (typhoon); 2 d (tropical storm); 0 d (> 100 mm precipitation)	↑ OR = 2.30 (other diarrheal diseases: ↑ OR = 3.56)	N/A
Fredrick (2015) [27]	Case-control	Cyclone Thane/ 2011	India	Cholera	10 d	+AR = 11%	CFR: 0.1%
Ni (2014) [28]	Retrospective	Unknown /2004–2010	China	Bacillary dysentery	0–1 mo	N/A	↑ RR of morbidity: 1.55 (moderate floods); ↑ 1.74 (severe floods)
Liu, Z (2015) [29]	Retrospective	Unknown/2010–2014	China	Bacillary dysentery	0–1 mo	↑ RR = 1.44	Average annual YLD/1,000 = 0.009
Liu, Z (2016) [30]	Retrospective	Unknown /2005–2011	China	Bacillary dysentery,	0–3 wk	↑ RR = 1.32 (lag 1 wk); ↑ Cumulative RR = 1.52 (lag 0–2 wk)	N/A
Na (2016) [31]	Retrospective	Unknown /2002–2012	South Korea	Bacillary dysentery, typhoid/ paratyphoid fever	2 wk (bacillary dysentery); unidentified (typhoid/ paratyphoid fever)	↑ RR = 3.10 (bacillary dysentery); ↔ typhoid and paratyphoid fever	N/A
Zhang (2016) [32]	Case-crossover	Unknown /2005–2011	China	Bacillary dysentery	4–12 d	↑ RR = 1.41 (lag 7 d); ↑ RR = 1.42 (lag 11 d)	N/A
Zhang (2016) [33]	Retrospective	Unknown/2007	China	Bacillary dysentery	1–3 d	↑ OR = 1.85	N/A
Liu (2017) [34]	Retrospective	Unknown/2004–2010	China	Bacillary dysentery	N/A	↑ RR = 1.17 (moderate floods); ↑ RR = 1.39 (severe floods)	N/A
Xu (2017) [35]	Retrospective	Unknown/2004–2010	China	Bacillary dysentery	2 wk	↑ RR = 1.17; *r* = 0.182 at wk 2	Weekly morbidity (per 100,000): 0.87
Denue (2018) [36]	Retrospective	Unknown/2017	Nigeria	Cholera	N/A	+AR (per 100,000) = 395.3	Overall CFR: 0.87%
Hu (2018) [37]	Retrospective	Unknown/2005–2009	China	Bacillary dysentery	0 d	↑ RR = 2.80	Overall attributable YLD: 1.87 per 1,000
Rieckmann (2018) [38]	Retrospective	Unknown/1990–2010	Sub-Saharan African countries	Cholera	N/A	↑ IRR = 144	N/A
Cambaza (2019) [39]	Observational	Cyclone Kennet/ Apr 2019	Mozam- bique	Cholera	1–2 wk	- + AR (per 100,000) = 98.7	no death
Ding (2019) [11]	Retrospective ecological	Unknown/2005–2012	China	Bacillary dysentery	5 d	↑ OR = 1.27	N/A
Gong (2019) [40]	Retrospective	Unknown /2013–2017	China	Infectious diarrhea	0–7 d	↑ RR = 1.05 (moderate flood); ↑ RR = 1.04 (severe flood)	N/A
Colston (2020) [41]	Observational	Unknown/2011–2012	Peru	ETEC infection, adenovirus infection, rotavirus infection, sapovirus infection, bacillary dysentery, *Campylobacter* infection	Within wk	- Early flood period ↑ RR = 1.73 (ETEC infection) ↑ RR = 0.36 (adenovirus infection) - Late flood period ↑ RR = 5.30 (rotavirus infection) ↑ RR = 2.47 (sapovirus infection) ↑RR = 2.86 (bacillary dysentery) ↑ RR = 1.41 (*Campylobacter* infection)	N/A
**Studies Reporting Absolute Outcome Measures**							
Natuzzi (2016) [42]	Observational	Unknown/2014	Solomon Island	Non-specific diarrhea	5 wk	+ 164.9 cases/week (2014) vs. 2013	+ 375% BOR; 10 deaths
Zheng (2017) [43]	Retrospective	Unknown/2005–2011	China	Bacillary dysentery, paratyphoid fever	N/A	↑ Bacillary dysentery; ↑ paratyphoid fever; ↑other infectious diarrhea (effect size: N/A)	N/A
Hulland (2019) [44]	Retrospective	Hurricane Matthew/ 2016	Haiti	Cholera	N/A	↑ Mean cases/d: 138.4 vs. 92.3 (post- vs. pre-hurricane)	↑Mean deaths/day: 2.4 vs. 1.1 (post- vs. pre-hurricane)
Birhan (2023) [45]	Observational study	Overflow of the Ribb River/ Mar 2021	Ethiopia	Non-specific diarrhea in children < 5 years	N/A	+ P: 29% vs. 12% (baseline)	N/A
Bwire (2023) [46]	Observational	Unknown /2019	Uganda	Cholera	2 wk	+ Cases: 67	CFR 1.5%
Luo (2023) [47]	Retrospective	Unknown /2016–2020	China	Typhoid/ paratyphoid fever, bacillary dysentery	1 d (typhoid/ paratyphoid fever); 3 d (bacillary dysentery)	↑ Inc (per 100,000): 0.3 vs. 0.1 (flood vs. non-flood) (typhoid/paratyphoid fever) ↑ Inc (per 100,000): 2.0 vs. 0.8 (flood vs. non-flood) (bacillary dysentery)	N/A
Lynch (2023) [48]	Retrospective	Tropical Cyclonic Storms/1996–2018	USA	Cryptosporidiosis, STEC	1–2 wk	+ Cases: 52% (cryptospiridiosis); + cases: 48% (week 1), 33% (week 2) (STEC)	N/A
Nisar (2024) [49]	Observational	Unknown/2022	Pakistan	Cholera	6 wk	+ Cases: 14,283	41 deaths
Sajid (2023) [50]	Observational	Unknown/2022	Pakistan	Bacillary dysentery, typhoid fever, cholera	N/A	*P* = 8% (bacillary dysentery); *P* = 10% (typhoid fever); *P* = 7% (cholera)	N/A

Abbreviations: ↑: Statistically significant increase; +: Increase; ↔: No change; AR: attack rate, BOR: bed occupying rate; CFR: case fatality rate; d: day(s); ETEC: Enterotoxigenic *Escherichia coli*; Inc: incidence; IRR: incidence rate ratio; mm: millimeter; mo: month(s); OR: odds ratio; N/A: not available; P: prevalence; r: Pearson correlation coefficient; RR: risk ratio; wk: week(s); STEC: Shiga toxin-producing *Escherichia coli*; YLD: years of healthy life lost due to disability

**Table 3:** Summary of reported outbreak patternd and epidemiology on reported hepatitis A/E cases

Study (year)	Study Design	Flood Description (Cause/Year)	Location Country (city)	Disease(s)	Lag Period (d/wk/mo)	Epidemiological Outcome	Impact on Health Service
**Studies Reporting Relative Effect Estimates**							
Gao (2016) [14]	Observational	Unknown/2007	China	Hepatitis A/E	15 d (hepatitis A); 18 d (hepatitis E)	Inc (per 100,000): 0.315 (hepatitis A); ↑ OR = 1.40 (hepatitis A) ↔ hepatitis E	N/A
Gao (2016) [51]	Retrospective	Unknown/2005–2010	China	Hepatitis A	24 d	↑ OR = 1.28	N/A
**Studies Reporting Absolute Outcome Measures**							
Pal (2016) [52]	Observational	Himalayan Tsunami /2013	India	Hepatitis A	N/A	+ Seroprevalence: 23/25 (92%)	No death
Zheng (2017) [43]	Retrospective	Unknown / 2005–2011	China	Hepatitis A	N/A	↑ Cases (effect size: N/A)	N/A
Silveria (2021) [53]	Retrospective	Unknown/2013	Brazil	Hepatitis A	4–8 wk	↑ Cases: + 300%	N/A
Nisar (2024) [49]	Observational	Unknown/2022	Pakistan	Hepatitis A/E	N/A	+ P: 18.7%	N/A

Abbreviations: ↑: Statistically significant increase, +: Increase, ↔: No change, d: day(s), Inc: incidence, N/A: not available, OR: odds ratio, P: prevalence, wk: week(s)

**Table 4:** Summary of reported outbreak patternd and epidemiology on reported other waterborne infections cases

Study (year)	Study Design	Flood Description (Cause/Year)	Location Country (city)	Disease(s)	Lag Period (d/wk/mo)	Epidemiological Outcome	Impact on Health Service
**Studies Reporting Relative Effect Estimates**							
Bloom (2016) [54]	Retrospective	Hurricane Sandy /2012	USA	Waterborne infections	N/A	↔ RR = 0.58 (OPD)	N/A
Na (2016) [31]	Retrospective	Unknown/ 2002–2012	South Korea	VVS	2–3 wk	↑RR = 2.49 (wk 2); ↑RR = 2.01 (wk 3)	N/A
**Studies Reporting Absolute Outcome Measures**							
Ito (2015) [55]	Observational	Unknown/ 2012	Nigeria	Schistosomiasis	N/A	↑P: 5.49% (IDP) vs. 0% (control)	N/A
Amarnath (2016) [56]	Observational	Unknown /2015	India	Waterborne infections	N/A	P: 19.2%	N/A
Lynch (2022) [57]	Retrospective	Unknown/ 2000–2011	USA	Legionnaires’ disease	N/A	N/A	Hospitalization: + 32%
Lynch (2023) [48]	Retrospective	Tropical Cyclonic Storms/ 1996–2018	USA	Legionnaires’ disease	2 wk	+P: 42%	N/A
Zaghi (2024) [58]	Case series	Unknown/ 2023	Italy	Non-cholera *Vibrio* spp. invasive infection	15 wk	+ Cases: 5 (baseline: 1/yr)	2/5 deaths

Abbreviations: ↑: Statistically significant increase, +: Increase, ↔: No change, IDP: Internally Displaced Persons, N/A: not available, RR: risk ratio, P: prevalence, STEC: Shiga toxin-producing *Escherichia coli*, USA: United States of America, VVS: *Vibrio vulnificus* septicemia, wk: week(s)

**Table 5:** Summary of reported outbreak patternd and epidemiology on reported dengue cases

Study	Study Design	Flood Description (Cause/Year)	Country	Lag Period(d/wk/mo)	Epidemiological Outcome	Impact on Health Service
**Studies Reporting Relative Effect Estimates**						
Zheng (2017) [43]	Retrospective	Unknown/2005–2011	China	N/A	↑ RR (effect size: N/A)	
Li (2021) [59]	Retrospective	Unknown /2013–2018	China	5–6 d	↑ RR = 1.62	N/A
Li (2022) [60]	Retrospective	Unknown/2015–2019	China	0–1 wk	↑ RR = 1.41	N/A
**Studies Reporting Absolute Outcome Measures**						
Aumentado (2015) [61]	Observational	Typoon Haiyan /2013	Phillipines	N/A	(-)Cases: 167 vs. 5,216 (2013 vs. 2014)	CFR 0.4%
Gao (2016) [14]	Observational	Unknown/2007	China	N/A	No reported cases	N/A
Natuzzi (2016) [42]	Observational	Unknown/2014	Solomon Island	N/A	↓ Cases: 346 vs. year 2013	N/A
Chang (2016) [62]	Retrospective	Typoon Haiyan /2013	Phillipines	N/A	N/A	↑ Hospitalization: 17 vs. 3 (post- vs. pre-flood)
Harris (2024) [63]	Observational	Cyclone Yaku /2023	Peru	3–4 wk	67% of cases attributable to cyclone from overall dengue infection	N/A
Nisar (2024) [49]	Observational	Unknown/2022	Pakistan	N/A	+ Cases: 8,906	42 deaths

Abbreviations: ↑: Statistically significant increase, ↓: Statistically significant decrease, (-): decrease, CFR: case fatality rate, d: day(s), N/A: not available, P: prevalence, RR: risk ratio, wk: week(s)

**Table 6:** Summary of reported outbreak patternd and epidemiology on malaria cases

Study (year)	Study Design	Flood Description (Cause/Year)	Location Country (city)	Lag Period (d/wk/mo)	Epidemiological Outcome	Impact on Health Service
**Studies Reporting Relative Effect Estimates**						
Ding (2014) [64]	Case-crossover	Unknown/ 2007	China	25 d	↑ aHR: 1.467	YLDs per 1000: 0.009/ d
Boyce (2016) [65]	Quasi-experimental observational	Unknown/2013	Uganda	3 mo	↑ *P. falciparum* test positivity rate: 39.4% vs. 25.8% (post vs. pre-flood); aRR: 1.47	↑ Hospitalizations: 781vs 504 (post vs. pre-flood) aIRR: 1.40
Gao (2016) [14]	Observational	Unknown/2007	China	27 d	+ Inc (per 100,000): 17.87 ↑ OR = 3.44	N/A
Chirebvu (2016) [66]	Retrospective	Unknown/ 2005–2010	Botswana	0 mo (flood discharge) 6 mo (flood extent)	↑ Flood discharge: *r* = 0.396; ↑ Flood extent: *r* = 0.467	N/A
Zheng (2017) [43]	Retrospective	Unknown/ 2005–2011	China	N/A	↓ RR (effect size: N/A)	N/A
Elsanousi (2018) [67]	Retrospective	Unknown/ 2013	Sudan	N/A	↑ IR (per 100,000 person-days): 6.09 (2011); 6.48 (2012); 8.24 (2013)	↑ Cases in OPD: 19.7% (2013) vs. 12.85% (2011) vs. 12.16% (2012)
Balikuddembe (2023) [68]	Retrospective	Unknown/ 1990–2019	Ethiopia, Kenya, Somalia, Sudan, and Tanzania	N/A	- Flood occurence ↓ *r* = − 0.586 (Kenya); ↓ *r* = − 0.421 (Somalia) - Flood duration ↑ *r* = 0.294 (Ethiopia); ↓ *r* = − 0.657 (Kenya); ↓ *r* = − 0.420 (Sudan); ↓ *r* = − 0.457 (Tanzania)	N/A
Xu (2024) [69]	Observational	Unknown/2020	Uganda	N/A	- ↑RR = 2.41 (mth 1), 7.61 (mth 2), 5.57 (mth3) post-flood	N/A
**Studies Reporting Absolute Outcome Measures**						
Natuzzi (2016) [42]	Observational	Unknown/2014	Solomon Island	N/A	↓ Cases: 2,667 cases (vs. 2013)	N/A
Chan (2017) [70]	Observational	Tropical cyclone Pam/2015	Vanuatu	N/A	No outbreak	N/A
Sajid (2023) [50]	Observational	Unknown/2022	Pakistan	N/A	+ P: 44%	N/A
Nisar (2024) [49]	Observational	Unknown/2022	Pakistan	3 mo	+ Cases: 188,507	336 deaths

Abbreviations: ↑: Statistically significant increase; ↓: Statistically significant decrease; aHR: adjust hazard ratio; aRR: adjusted rate ratio; CFR: case fatality rate; d: day(s); IR: incidence rate; aIRR: adjusted incidence rate ratio; mo/mth: month(s); OPD: outpatient department; N/A: not available; P: prevalence; *P. falciparum*: *Plasmodium falciparum*; r: Pearson correlation coefficient; RDT: rapid diagnostic test; RR: risk ratio, YLDs: years of healthy life lost due to disability

**Table 7:** Summary of reported outbreak patternd and epidemiology on reported other vector-borne infections cases

Study (year)	Study Design	Flood Description (Cause/Year)	Location Country (city)	Disease(s)	Lag Period (d/wk/mo)	Epidemiological Outcome	Impact on Health Service
**Studies Reporting Relative Effect Estimates**							
Gao (2016) [14]	Observational	Unknown/2007	China	JE	11 d	Inc (per 100,000): 0.34; ↔ OR = 0.58	N**/**A
Zhang (2016) [71]	Case-crossover	Unknown/ 2007–2012	China	JE	23 d	↑OR = 2.00	N/A
Ding (2019) [11]	Retrospective ecological	Unknown/ 2005–2012	China	JE	0 d	↑OR = 2.33	N**/**A
Tall (2020) [72]	Retrospective	Unknown / 1991–2013	Australia	RRV disease	1–3 mo	↑ OR = 10.8 (North-west Slopes); 3.6 (North-west Plains); 9.1 (West Murray)	N/A
**Studies Reporting Absolute Outcome Measures**							
Amarnath (2016) [56]	Observational	Unknown/2015	India	Vector- borne infections	N/A	P: 13.8%	N/A
Adekunle (2019) [73]	Mathematical modeling	Unknown/2009	Australia	RRV disease, Barmah Forest Virus disease	2–3 wk (predicted)	Predicted cases: 96–104 vs. 30 (baseline)	N/A

Abbreviations: ↑: Statistically significant increase, ↔: No change, d: day(s), JE: Japanese encephalitis, mo: month(s), OR: odds ratio, N/A: not available, P: prevalence, RRV: Ross River virus, wk: wee

### Study outcomes

The primary outcomes of this systematic review were to evaluate (1) flood characteristics and lag periods of waterborne or vector-borne infections (2) the incidence, prevalence, or association between flooding and waterborne or vector-borne infections; (3) mortality, morbidity, and other health outcomes associated with waterborne or vector-borne infections; and (4) prevention and response measures of waterborne and vector-borne infections related to flooding.

## Results

### Study selection

**Fig. 1:**
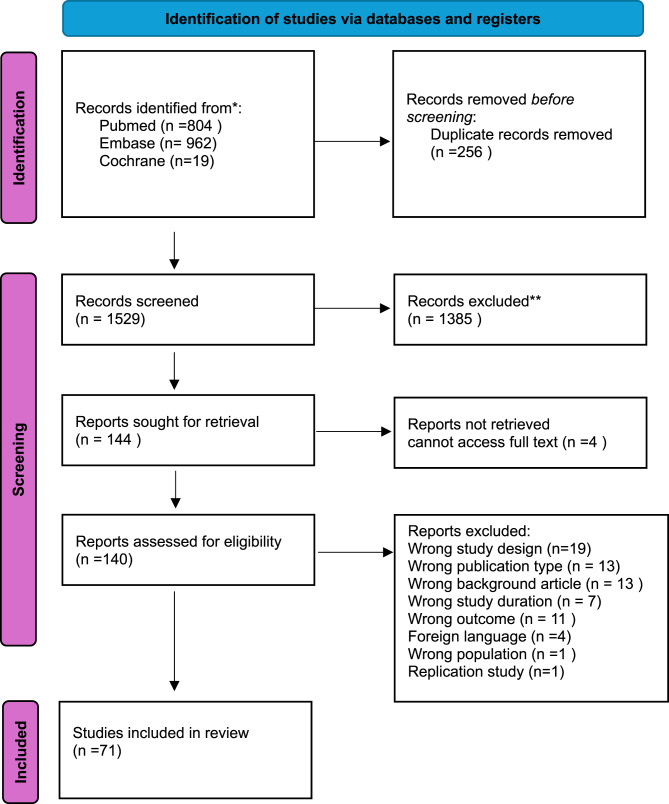
PRISMA 2020 flow diagram of study selection

A total of 1785 records were identified through database searching. After removing duplicates and screening titles and abstracts, 140 full-text articles were assessed for eligibility. Of these, 71 studies met the inclusion criteria and were included in the final review. The study selection process is illustrated in the PRISMA flow diagram (Fig. 1).

### Study characteristics

The included studies were conducted between 2014 and 2024 across 30 countries, primarily in Africa (*n* = 9), Asia (*n* = 9), the Americas (*n* = 4), Europe (*n* = 4), and Oceania (*n* = 4). These countries can be categorized into high-income countries (*n* = 15) and low- and middle-income countries (*n* = 15) according to the World Bank country income classifications for FY2025 [74]. The majority were retrospective studies (*n* = 34), followed by observational studies (*n* = 25), case-crossover studies (*n* = 5), case series (*n* = 3), case-control studies (*n* = 2), case report (*n* = 1), and non-randomized controlled study (*n* = 1). Flooding events investigated included monsoon floods, typhoons, cyclones, and urban flash floods. The most frequently studied disease was infectious diarrhea (*n* = 30), followed by leptospirosis (*n* = 18), malaria (*n* = 14), dengue (*n* = 9), hepatitis A/E (*n* = 6), and other waterborne and vector-borne infections (*n* = 14). A summary of study characteristics is presented in Tables 1, 2, 3, 4, 5, 6 and 7.

### Overall risk of bias findings

Of the 71 included studies, 17 (26%) were rated as low risk, 45 (63%) as moderate risk, and 8 (11%) as high risk of bias. The most frequent methodological shortcomings were incomplete reporting of key outcomes, insufficient detail on exposure assessment (particularly the characterization of flood severity and duration), and reliance on retrospective data sources without adequate control for confounding. Higher-quality studies tended to report more consistent associations between flooding and infectious disease outcomes, while studies with moderate to high risk of bias frequently presented outcomes as simple counts or crude proportions without statistical comparisons. This limited the precision and interpretability of their findings and made it more difficult to integrate these results into the overall synthesis.

## Impact of flooding on waterborne infections

Evidence from included studies suggests an association between flooding and increased transmission of waterborne infections; however, findings should be interpreted cautiously in light of the predominance of observational designs and moderate risk-of-bias ratings. The magnitude and consistency of reported associations varied across pathogens and settings. Diseases are grouped based on similarities in transmission mechanisms, incubation periods, epidemiological patterns, and prevention strategies. Leptospirosis is grouped separately due to its unique environmental reservoirs, animal-to-human transmission, moderate incubation period, and the availability of chemoprophylaxis. Infectious diarrhea (e.g., cholera, bacillary dysentery, typhoid/paratyphoid fever, and cryptosporidiosis) forms another category characterized by fecal-oral transmission, rapid onset following floods, and reliance on hygiene-based control measures. Hepatitis A/E share prolonged incubation periods and vaccine-driven prevention opportunities, constituting a distinct third group. Lastly, less commonly reported but clinically significant pathogens such as *Legionella* spp., non-cholera *Vibrio* spp., and *Schistosoma* spp. are described under “other waterborne infections,” reflecting their distinct ecological niches and specific public-health implications.

### Leptospirosis

A total of 16 studies reported on leptospirosis outbreaks associated with flooding across diverse geographic settings, including Australia, Bosnia and Herzegovina, Fiji, Greece, India, Japan, Malaysia, Puerto Rico, Taiwan, Thailand, and the United States of America (USA) (Table 1). The study designs included observational studies (*n* = 6), retrospective studies (*n* = 7), case series (*n* = 2), and case report (*n* = 1).

#### Epidemiologic associations between flooding and infection outcomes

##### Flood characteristics and lag periods

Most studies described floods caused by tropical cyclones, typhoons, or monsoonal rains. The reported lag periods between flood events and increased leptospirosis cases ranged from 1 to 12 weeks, with peaks commonly observed at 2–6 weeks post-flooding.

##### Outbreak patterns and epidemiology

Among the 16 studies identified, most reported increased leptospirosis incidence following flooding, although the magnitude and precision of effect estimates varied across settings (Table 1). Factors associated with these outbreaks included not only the severity and duration of flooding but also environmental and exposure-related risks such as proximity to rivers and garbage sites, and contact with animals, including rodents, pigs, and cattle. These associations were supported by genotypic typing and spatial analysis [10, 12, 15].

Leptospirosis cases have also been reported in non-endemic regions following flooding. In Japan, a 69-year-old man in Hiroshima Prefecture developed leptospirosis two weeks after typhoon-related flooding in 2022, despite no travel to endemic areas. Diagnosis was confirmed by microscopic agglutination test (MAT), identifying *Leptospira interrogans* serovar *Rachmati* [20]. In the U.S. Virgin Islands, the first human cases were identified after Hurricanes Irma and Maria in 2017. All had flood-related exposure [16]. A case series in Greece reported 13 confirmed cases following Storm Daniel in 2023. All individuals had direct floodwater contact, with most exposures occurring during household or community cleanup efforts [22]. However, a serological survey from Taiwan and an epidemiological study from Anhui, China, found no evidence of leptospirosis outbreaks after flooding [14, 75].

#### Health system impact

Evidence regarding the health system burden of flood-associated leptospirosis remains limited and variable. Increased hospitalization rates for leptospirosis following flooding were reported in 2 studies, ranging from 69% to 72% [19, 24]. However, Jones et al. observed a lower hospitalization rate post-flooding, 72% compared to 93% during non-flood periods, possibly reflecting increased detection of less severe cases, earlier initiation of treatment, or both [24]. Case fatality rates ranged from 0.5% to 6% in most post-flood leptospirosis outbreaks, with sporadic higher mortality reported in regions experiencing severe flooding and delayed access to healthcare [15, 24]. Aside from hospitalization rates, other indicators of healthcare impact, such as intensive care unit admissions, length of hospital stay, or healthcare resource utilization, were rarely reported across studies.

#### Prevention and response intervention

Only two studies have evaluated doxycycline chemoprophylaxis for preventing leptospirosis following flood exposure. Both were observational in design, which limits causal interpretation; however, the findings consistently suggested a protective effect (Table S4). In southern Thailand (2010), a nonrandomized controlled study reported that a single 200 mg dose of doxycycline achieved 76.8% protective efficacy (risk ratio [RR] = 0.23; 95% CI: 0.08–0.66), though protection decreased with repeated floodwater exposure [76]. In Mumbai, India (2017), a large-scale chemoprophylaxis campaign was implemented using doxycycline for adults and azithromycin for children and pregnant women, with dosing stratified by risk level (low risk: single dose of doxycycline 200 mg or azithromycin 500 mg; moderate risk: doxycycline 200 mg once daily for 3 days; and high risk: doxycycline 200 mg once weekly for 6 weeks). Supported by public health messaging and inter-agency coordination, the intervention reduced confirmed leptospirosis cases to 59 in 2017, compared to 432 cases during the 2005 floods without chemoprophylaxis [77]. Although these findings suggest potential benefit, robust comparative trials are lacking, and the overall certainty of evidence remains low.

### Infectious diarrhea

A total of 31 studies reported on infectious diarrhea outbreaks associated with flooding across diverse geographic settings, including China, Ethiopia, Haiti, India, Mozambique, Nigeria, Peru, sub-Saharan African countries, and Uganda. (Table 3). The study designs included retrospective studies (*n* = 17), observational studies (*n* = 9), case-control studies (*n* = 2), and case-crossover studies (*n* = 3).

#### Epidemiologic associations between flooding and infection outcomes

##### Flood characteristics and lag periods

The reported lag periods ranged from same-day effects to six weeks post-flooding. Specifically, same-day to 14-day lag periods were observed for typhoid/paratyphoid fever, bacillary dysentery, and amoebiasis in several studies from China and South Korea [11, 14, 26, 28–30, 32, 33, 35, 37, 41, 42, 47, 49, 78] while cholera typically showed a lag period of 1–2 weeks, cryptosporidiosis exhibited a longer lag of 6 weeks [27, 39, 46, 79]. In contrast, infections such as rotavirus infection, sapovirus infection, shigellosis, and *Campylobacter* infection were more commonly associated with the later phase, occurring 15–21 weeks post-flooding [41].

##### Outbreak patterns and epidemiology

Evidence from 31 studies across diverse settings suggests an overall increase in diarrheal incidence following flooding; however, statistically significant post-flood elevations were observed only for selected pathogens, and effect sizes varied in magnitude and precision across studies. (Table 2). The most consistent increases were observed in bacterial infections such as cholera, bacillary dysentery, with additional rises reported for several viral and protozoal infections. In contrast, findings for typhoid and paratyphoid fever were mixed, with one study reporting no clear association with flooding [14], while another showed an increase in incidence during the post-flood period [43].

Cholera was reported in multiple studies using various measures such as attack rate, incidence rate ratio, or raw case counts; however, all consistently demonstrated an increase following flooding [25, 36, 39, 44]. Similarly, bacillary dysentery showed post-flood increases, with reported risk ratios ranging from 1.17 (95% CI: 1.03–1.33) to 2.86 (95% CI: 1.81–4.52) [26, 28, 30, 33–35, 37, 78]. In Peru, one study further reported that the post-flood period was associated with increased risks of multiple enteric pathogens, including rotavirus (RR = 5.30; 95% CI: 2.70–10.40), sapovirus (RR = 2.47; 95% CI: 1.79–3.41), *Shigella* spp. (RR = 1.73; 95% CI: 1.10–2.71), and *Campylobacter* spp. (RR = 1.41; 95% CI: 1.01–1.97) [41]. In Taiwan, a post-flood serological survey conducted after Typhoon Morakot found a sharp increase in recent *Entamoeba histolytica* infection, with seroconversion rates reaching up to 40% [75]. An outbreak of cryptosporidiosis was reported following the 2013 flooding of the River Saale in Germany. Case-control analysis showed a higher infection risk among individuals who visited floodplain areas (Odds ratio [OR] = 5.5; 95% CI: 1.40–21.56). *Cryptosporidium* oocysts were detected in public swimming pools, the river, and floodplain waters, indicating widespread water contamination [79].

Several studies examined how specific flood characteristics influenced bacillary dysentery risk, with eleven reporting positive associations [11, 26, 29–35, 37, 41]. Flood severity was consistently associated with higher bacillary dysentery risk, with Ni et al. reporting a 55% increase during moderate floods (RR = 1.55; 95% CI: 1.42–1.67) and 74% during severe floods (RR = 1.74; 95% CI: 1.56–1.94) [28]. Similarly, Gong et al. observed increased risk with both moderate (RR = 1.05; 95% CI: 1.02–1.09) and severe floods (RR = 1.04; 95% CI: 1.01–1.08) [40]. In contrast, the impact of flood duration on bacillary dysentery morbidity was inconsistent. Ni et al., Liu X et al., and Hu et al. reported a negative correlation, suggesting that short but intense floods had a greater impact than prolonged flooding [28, 37, 78]. Conversely, Liu Z et al. found that the risk increased with each additional day of flooding (RR = 1.08; 95% CI: 1.04–1.12) [29].

#### Health system impact

The burden of infectious diarrhea outbreaks, measured by Years Lived with Disability (YLDs), was reported to increased during flood periods [29, 78]. Hospitalizations also rose, with the Solomon Islands reporting a 375% increase in bed occupancy following flooding, resulting in staff shortages and early depletion of intravenous fluids [42]. Children and the elderly were the most affected groups, experiencing significantly elevated morbidity and mortality rates [27, 40, 43, 44]. Case fatality rates (CFRs) for cholera ranged from 0% to 5.1% [27], while mortality data for other diarrheal diseases were not reported.

#### Prevention and response intervention

No studies have specifically evaluated the role of antibiotic prophylaxis in preventing diarrheal diseases in the context of flooding. Vaccination efforts have primarily targeted cholera; however, strong evidence on the effectiveness of oral cholera vaccine (OCV) in flood-related outbreaks remains limited, with most available data derived from observational studies. For example, the study by Cambaza et al. on the 2019 Cyclone Kenneth outbreak in Mozambique demonstrated that combining two does of OCV with early public health messages, quick identification of cases, and fast setup of treatment facilities was essential for effectively controlling the outbreak [39]. Similarly, in Uganda, rapid OCV deployment achieved over 90% coverage and ended the outbreak within 10 weeks [46].

Delayed detection and limited access to treatment were associated with increased cholera mortality following flooding. During Nigeria’s 2010 floods, these factors contributed to a high case fatality rate (CFR) of 14.5% among individuals aged ≥ 65 years [25]. By comparison, the 2017 floods saw a markedly lower CFR of 0.87%, attributed to improved surveillance, rapid response, and timely treatment [36]. Overall, although these experiences suggest potential benefit from timely vaccination and coordinated response measures, the certainty of evidence remains limited.

### Hepatitis A/E

A total of 6 studies reported on hepatitis A, and 2 studies reported on hepatitis E associated with flooding events across diverse settings, including Brazil, China, India, and Pakistan. (Table 3). The study designs included retrospective (*n* = 3) and observational (*n* = 3) studies.

#### Epidemiologic associations between flooding and infection outcomes

##### Flood characteristics and lag periods

The cause of flooding was not specified in most studies. The reported lag period for hepatitis A ranged from 2 to 8 weeks post-flooding, while the lag periods of hepatitis E was 18 days.

##### Outbreak patterns and epidemiology

Of the 6 studies reviewed, only 4 specified outcome measures. Flooding was reported to be associated with an increase in hepatitis A cases ranging from 18% to 300% [49, 53], with reported odds ratios ranging from 1.28 (95% Cl: 1.05–1.55) to 1.40 (95% Cl: 1.17–1.77) [14, 51]. The severity of flooding appeared to influence infection risk. A study from China found that only severe floods were significantly associated with an increased incidence of hepatitis A (OR = 1.28; 95% CI: 1.05–1.55), while moderate and mild floods showed no significant association [51]. In addition, outbreaks were often linked to poor sanitation and overcrowding, particularly in post-disaster settings such as flood rescue camps, where disruption of water supplies, fecal contamination, and limited access to hygiene facilities may further facilitate hepatitis A virus transmission [52]. In contrast, no significant association was found between flooding and hepatitis E (OR = 0.94; 95% CI: 0.7–1.31); however, this finding was based on a single study [14].

#### Health system impact

Although flooding was reported to be associated with an increase in hepatitis A incidence, mortality appeared low; however, available data on mortality were limited [52]. However, no study provided detailed information on health system impacts such as hospitalization rates, disease severity, or healthcare resource utilization related to flood-associated hepatitis A or hepatitis E.

#### Prevention and response intervention

No studies reported on specific prevention or response measures for hepatitis A or hepatitis E in the context of flooding.

### Other waterborne infections

Aside from leptospirosis, infectious diarrhea, and hepatitis A/E, other waterborne infections were also reported following flooding (Table 4). A total of 7 studies documented such infections across various countries, including Nigeria, Taiwan, the USA, South Korea, and China. The study designs included retrospective studies (*n* = 4), observational studies (*n* = 2), and case series (*n* = 1).

#### Epidemiologic associations between flooding and infection outcomes

##### Flood characteristics and lag periods

Most flooding events occurred following typhoons, hurricanes, or tropical cyclonic storms. The reported pathogens included *Legionella* spp., non-cholera *Vibrio* spp. and *Schistosoma* spp. Lag period varied by pathogen: *Vibrio vulnificus* septicemia peaked 2–3 weeks post-flooding in South Korea and up to 15 weeks in Italy [31, 58]; and Legionnaires’ disease appeared around 2 weeks post-flooding [48].

##### Outbreak patterns and epidemiology

Although no study directly reported the incidence of Legionnaires’ disease following flooding, the consistent rise in hospitalizations suggests an increase in disease occurrence during post-flood periods. In the United States, hospitalization rates due to Legionnaires’ disease increased by 32–54% during flood events between 1996 and 2018, particularly two weeks after major storms, with elevated rates persisting into the third week [48, 57].

Non-cholera *Vibrio* infections are another important group of waterborne diseases to monitor in the aftermath of flooding. In South Korea the incidence of *Vibrio vulnificus* septicemia increased significantly following meteorologic disasters between 2002 and 2012, peaking in the second week post-flood (RR = 2.49; 95% CI: 1.47–4.22), remaining elevated in the third week, and returning to baseline by the fourth week [31]. Similarly, Zaghi et al. reported five new cases of invasive *Vibrio vulnificus* infection following flooding [58].

In addition to bacterial infections, parasitic diseases may also re-emerge in flood-affected endemic regions. An increased prevalence of schistosomiasis was observed during the 2012 flood in Nigeria, with a significantly higher rate in the exposed group (5.49%) compared to the control group (0%) [55]. The use of unsafe water, poor hygiene practices, and low caregiver education were reported to be significantly associated with higher infection rates.

#### Health system impact

Limited data are available on health system impacts related to other waterborne infections following flooding. Where available, data indicated notable strain on healthcare services. In the United States, floods were associated with a 32–54% increase in Legionnaires’ disease hospitalizations [48]. For *Vibrio vulnificus* infections, although hospitalization and ICU admission rates were not consistently detailed, case series data highlighted a high case fatality rate (40%) [58], indicating severe clinical outcomes.

#### Prevention and response intervention

No studies reported on specific prevention or response measures for these infections in the context of flooding.

### Impact of flooding on vector-borne diseases

Flooding influences vector-borne disease transmission through altered vector habitats and environmental conditions [65]. In this section, vector-borne infections are categorized into three groups: dengue infection, malaria infection, and other vector-borne infections. The classification is based primarily on epidemiological patterns and the number of studies identified.

### Dengue

A total of 9 studies reported on dengue associated with flooding (Table 5). These studies were conducted in China, Pakistan, Peru, the Philippines, and the Solomon Islands. The study designs included retrospective studies (*n* = 4) and observational studies (*n* = 5).

#### Epidemiologic associations between flooding and infection outcomes

##### Flood characteristics and lag periods

Most flooding occurred by typhoons or cyclones, that occurred in Asia and South Africa. Outbreaks were reported early, with lag period ranging from 5 days to 3–4 weeks post-flooding.

##### Outbreak patterns and epidemiology

The impact of flooding on dengue outbreak differed across study locations, with some countries reporting significant increases while others observed no change or decline. In China post-flood dengue risk increased, with reported RRs ranging from 1.41 (95% CI: 1.17–1.69) to 1.62 (95% CI: 1.45–1.80) [59, 60]. Similarly, a study from the Philippines observed a significant increase in dengue cases in hospital post-flooding, rising from 3 cases before the flood to 17 cases afterward (*p* < 0.00001) [62]. In Pakistan, nearly 9,000 dengue cases were reported following the 2022 floods [49]. However, some studies documented either no outbreak or a decline in dengue incidence post-flooding [14, 42]. Entomological investigations in affected regions further revealed that discarded containers (50%) and tyres (18%) were the predominant mosquito breeding sites [61].

#### Health system impact

A study from the Philippines indicated that dengue outbreaks following flooding were associated with increased hospitalizations [62]. One study reported 42 deaths from dengue following flooding [49].

#### Prevention and response intervention

No randomized controlled trials were identified evaluating post-flood dengue prevention strategies. Available evidence is derived primarily from observational and field-based evaluations. Findings from cross-sectional and descriptive studies suggest that timely vector control and coordinated response measures may contribute to mitigating dengue transmission following flooding. For example, a study by Aumentado et al. reported that timely, coordinated interventions helped prevent a large dengue outbreak following a typhoon [61]. The response involved a multifaceted strategy implemented by national and international stakeholders under the coordination of the WHO and the Philippine Department of Health. Disease surveillance was rapidly re-established, with reporting frequency increased from weekly to daily within 10 days. Rapid diagnostic tests (RDTs) were distributed to improve case detection, and refresher training on dengue case management was provided. Vector control operations including fogging, larviciding with pyriproxyfen, and search-and-destroy activities were initiated 12 days post-typhoon, initially targeting hospitals, schools, evacuation centers, and public areas. However, as these interventions were implemented simultaneously and without a comparison group, the independent effectiveness of specific measures cannot be determined. No studies reported on the use or impact of dengue vaccination in post-flood outbreak settings. Overall, while coordinated response strategies appear promising, the certainty of evidence regarding their effectiveness remains limited.

### Malaria

A total of 12 studies reported on malaria associated with flooding (Table 6). These studies were conducted in Africa, China, and South Asia. The study designs included observational studies (*n* = 7), retrospective studies (*n* = 4), and a case–crossover study (*n* = 1).

#### Epidemiologic associations between flooding and infection outcomes

##### Flood characteristics and lag periods

Most outbreaks following flooding were reported in Africa and Asia, where these diseases are endemic. Compared to dengue, malaria outbreaks generally exhibited longer lag periods, ranging from 1 to 6 months post-flooding [64–66].

##### Outbreak patterns and epidemiology

A total 8 malaria studies reported increased incidence or case positivity rates following flooding [64, 65, 67]. In contrast, countries where malaria has been eliminated reported no post-flood outbreaks [70]. The implementation of effective vector control interventions during the post-flood period appeared to be a key factor contributing to the reduction in malaria incidence as demonstrated by findings from the Solomon Islands, where a decline in malaria incidence was observed after flooding due to the prompt implementation of post-flood vector control measures [42]. Additional outbreak-associated factors include flood discharge and flood extent, both of which have demonstrated positive associations with malaria transmission risk [66]. In contrast, the relationship between flood duration and malaria incidence remains inconsistent, with findings varying across different countries and ecological settings [68].

#### Health system impact

A few studies reported an increase in both outpatient department (OPD) visits, approximately 7%, and malaria-related hospitalizations, which rose by more than 50% following flooding [65, 67]. Data on mortality are limited, with only one study reporting high mortality, particularly among children [80].

#### Prevention and response intervention

To date, there are no well-conducted randomized controlled trials evaluating malaria prevention strategies in post-flood settings. In Uganda, Boyce et al. reported that three monthly rounds of dihydroartemisinin-piperaquine (DP) administered as chemoprevention in children ≤ 12 years were associated with a 53.4% reduction in malaria incidence compared with control villages over six months (adjusted rate ratio = 0.47; 95% CI: 0.34–0.62; *P* < 0.01) (Table S5) [81]. However, as this was not a randomized trial, residual confounding cannot be excluded. Observational data also suggest that timely vector control measures may be associated with reduced outbreak magnitude. In Kenya, delayed implementation of vector control measures, including indoor residual spraying, long-lasting insecticidal nets, and larviciding, during the 1997–1998 floods led to high malaria incidence and elevated child mortality. In contrast, early interventions in 2006 effectively prevented an epidemic [80]. Comparably, Chan et al. reported no malaria outbreak following Tropical Cyclone Pam in Vanuatu, which was attributed to a long-standing malaria elimination program [70]. The intervention package included a 9-week mass drug administration, distribution of insecticide-treated nets, introduction of larvivorous fish to breeding sites, and continuous community-based microscopy since 1991. Likewise, the study by Natuzzi et al. reported a decrease in the incidence of dengue and malaria following a flood compared to non-flood years, attributed to prompt and aggressive vector control measures [42]. In Uganda, Xu et al. found that, despite mass chemoprevention with dihydroartemisinin-piperaquine in children under 12, about 30% of those who tested positive by rapid diagnostic test (RDT) remained or became RDT-positive in subsequent rounds. Long-lasting insecticidal net use was significantly lower among RDT-positive children (52.6%) compared to RDT-negative children (68.8%; *p* = 0.02) [69]. Overall, although chemoprevention and coordinated vector control strategies appear promising in post-flood settings, the certainty of evidence remains limited due to the absence of randomized trials and the potential for confounding in observational studies.

### Other vector-borne infections

A total of 6 studies reported on other vector-borne infections, predominantly Japanese encephalitis (JE) and Ross River virus (RRV) disease (Table 7). These studies were conducted in Australia, China, and India. The study designs included observational studies (*n* = 2), retrospective studies (*n* = 2), a case–crossover study (*n* = 1), and a mathematical modeling study (*n* = 1).

#### Epidemiologic associations between flooding and infection outcomes

##### Flood characteristics and lag periods

JE showed a short lag period, between 0 and 23 days [11, 14, 71], whereas RRV disease demonstrated longer lag period, varying between 2 weeks and 3 months [72, 73].

##### Outbreak patterns and epidemiology

The association between Japanese encephalitis (JE) and flooding was inconsistent in 3 studies, with reported odds ratios ranging from 0.33 (95% CI: 0.47–0.71) to 2.33 (95% CI: 1.12–4.87) [11, 14, 71]. For RRV disease, which is endemic to Australia, a retrospective study reported an increase in RRV cases following flooding, with odds ratios for infection across different regions ranging from 3.6 (95% CI: 1.0–13.1) to 10.8 (95% CI: 3.2–36.2) [72].

#### Health system impact

No studies reported on the health impact of JE or RRV disease in the context of flooding.

#### Prevention and response intervention

No studies reported on prevention or response measures for JE or RRV disease in the context of flooding.

## Discussion

To our knowledge, this is the first systematic review to comprehensively examine the impact of flooding on both waterborne and vector-borne infectious diseases. Across diverse geographic and epidemiologic contexts, most included studies reported increased disease incidence following flooding events, particularly in endemic regions. However, the predominance of observational designs and methodological heterogeneity across studies necessitate cautious interpretation, as causal relationships cannot be definitively established. Patterns of post-flood transmission varied substantially according to pathogen biology, environmental persistence, baseline endemicity, and public health response capacity.

Flooding was frequently associated with rapid increases in waterborne infections, with reported lag periods influenced by environmental persistence, transmission routes, and incubation periods. Enteric bacteria such as *Shigella spp.*, *Salmonella spp.*, and *Entamoeba histolytica* exhibited short lag periods (0–14 days) due to rapid fecal–oral transmission and brief incubation times [82]. In contrast, hepatitis A and E demonstrated longer lag periods of 2–8 weeks, attributable to subclinical transmission and extended incubation, which delay outbreak detection [83]. Some infections, including leptospirosis, cryptosporidiosis, and Legionnaires’ disease, often showed lag periods exceeding their incubation times, reflecting prolonged environmental survival of the pathogens [84–86].

Vector-borne diseases generally display longer lag periods following flooding compared with waterborne infections. For dengue, the lag ranges from approximately 5 days to 3–4 weeks, slightly exceeding the typical breeding cycle of *Aedes* mosquitoes [87]. This interval may be influenced by prior water availability, as environmental conditions affect the formation and persistence of mosquito breeding sites [88]. Malaria typically shows a longer lag of 2–3 months, likely due to initial destruction of mosquito larval habitats by fast-moving floodwaters, followed by resurgence as stagnant water accumulates. Contributing factors include disrupted vector control, overcrowding, and inadequate housing infrastructure [64–66]. JE has been reported with lag periods ranging from 0 to 23 days; earlier spikes likely reflect amplification of infections from already-infected adult mosquitoes rather than new transmission cycles [11].

Interpretation of reported lag periods should therefore be approached with caution. Definitions of “lag” were not standardized across studies and variably referred to the time from flood onset to the first reported case, peak incidence, or statistically modelled delay parameters in time-series analyses. Given this heterogeneity, may not reliably represent precise or universally predictive intervals.

Socioeconomic conditions were consistently described as important contextual modifiers of disease patterns. In low- and middle-income countries (LMICs), enteric infections were commonly reported following flooding, likely reflecting sanitation disruption, contamination of drinking water, population displacement, overcrowding, and limited health system surge capacity [89–93]. Consistent with WHO data, diarrheal deaths remain disproportionately high in these regions, with global estimates suggesting that up to 69% of the worldwide diarrheal disease burden is attributable to unsafe WASH conditions [94, 95]. In high-income countries, severe epidemics were rare, and reported outbreaks more commonly involved pathogens linked to engineered water systems, such as *Legionella* or *Cryptosporidium*, reflecting vulnerabilities within complex infrastructure and greater surveillance capacity [93, 96–98].

Baseline endemicity and vector distribution further shaped post-flood outcomes of vector-borne diseases. Most mosquito-borne disease outbreaks following flooding have been reported in East Asia, Southeast Asia, and Africa, where these infections are typically endemic. Regions without established Aedes mosquito populations, such as inland areas of China, did not experience dengue outbreaks despite flooding compare with southern coastal province [99–101]. Conversely, Natuzi et al. documented a decreased in dengue incidence following flooding in the Solomon Islands compared to the previous year. This may reflect timely vector control or temporal artifacts related to preceding epidemic intensity and population immunity [42].

Beyond geographic context, flood characteristics—including severity, duration, and cumulative exposure—also modified disease risk. Although most studies reported increased leptospirosis risk after flooding; however, some studies reported no outbreak, possibly due to lower rainfall intensity or limited cumulative exposure [14, 75]. Similarly, several studies have demonstrated a positive correlation between flood severity and the incidence of bacillary dysentery and malaria [34, 66]. Although findings were inconsistent across studies, likely due to variation in rainfall intensity, infrastructure robustness, and methodological differences. Individual-level exposure factors including direct floodwater contact, open wounds, and proximity to livestock or rodents were particularly relevant for leptospirosis and explain emergence in previously non-endemic areas [102]. These observations underscore the need for multisectoral interventions, including environmental management, personal protective measures, and targeted public health messaging.

Although mosquito-borne diseases were frequently reported after floods, no post-flood outbreaks of other vectors (e.g., ticks, fleas) were documented. This may reflect true biological differences, as mosquitoes depend on aquatic habitats that expand during flooding, whereas terrestrial vectors may be displaced or destroyed [87, 103]. However, underreporting and limited surveillance cannot be excluded. Our review also identified several less commonly recognized but clinically important post-flood infections, including cryptosporidiosis, non-cholera Vibrio septicemia, Legionnaires’ disease, and schistosomiasis. These findings highlight the need for broader pathogen and vector surveillance in post-flood settings.

Observed increases in infectious disease incidence following flooding should be interpreted cautiously, as several confounding factors may influence these associations. Many included diseases exhibit strong seasonal patterns, and heavy rainfall independent of flooding may also enhance transmission. In addition, intensified post-disaster surveillance, population displacement, sanitation disruption, and baseline endemicity may contribute to observed trends. These contextual factors should be considered when interpreting flood–disease associations.

Despite the absence of high-quality evidence, existing studies consistently suggests that effective prevention measures can mitigate post-flood transmission of waterborne and vector-borne infections. However, concurrent implementation of multiple interventions and potential confounding limit attribution of effect to specific strategies. Preventive strategies should be tailored to the pathogen’s transmission route and the expected lag period. Immediate actions such as emergency WASH interventions, provision of safe water, establishment of oral rehydration centers, and targeted health communication are essential for short-lag infections. In contrast, infections with longer lag periods require sustained measures including water quality monitoring, latrine reconstruction, food-safety education, and appropriate vector control. Targeted interventions may include leptospirosis chemoprophylaxis, oral cholera vaccination, or post-flood antimalarial chemoprevention, although evidence remains limited for dengue and hepatitis A vaccination in post-disaster settings.

Data quantifying mortality, hospital burden, and economic impacts remain sparse, limiting accurate assessment of overall disease burden. Available evidence suggests substantial strain on healthcare systems and significant economic losses, as seen with cholera outbreaks in Africa and cost-of-illness estimates from Malawi, alongside documented financial impacts of dengue and malaria [104–107]. The limited evidence base may contribute to underinvestment in preparedness and response despite potentially high societal costs.

## Strengths and limitations

This review has several key strengths. It provides broad geographic coverage across both endemic and non-endemic regions and includes a wide range of waterborne and vector-borne infections. It also highlights important temporal patterns, particularly the lag period between flooding and disease onset, which is critical for outbreak prediction. This review also identifies effective prevention and response strategies, such as WASH interventions, vector control, and chemoprophylaxis. Additionally, the review addresses the impact of flood-related outbreaks on healthcare services, offering practical insights for preparedness and response planning.

This review has several limitations. First, the lack of standardized outcome definitions across studies made it challenging to compare results directly or conduct meta-analysis. Second, most of the included studies were observational or retrospective in nature, increasing the risk of selection bias, recall bias, and incomplete data. Third, many studies lacked detailed reporting on key outcomes such as hospitalization rates, case fatality, or healthcare system burden, limiting the ability to assess the full impact of outbreaks. Moreover, variations in study quality likely contributed to heterogeneity in findings; studies with stronger methodological rigor tended to report more precise effect estimates, whereas those with weaker designs often presented inconsistent or incomplete outcomes.

The search strategy was limited to PubMed, Embase, and the Cochrane Library, which provide comprehensive coverage of peer-reviewed biomedical and public health literature and are commonly recommended for systematic reviews in infectious diseases. However, restricting the search to these databases may have resulted in database bias, as relevant studies indexed in regional or multidisciplinary databases may not have been captured. The review was also restricted to English-language publications due to resource constraints and the absence of formal translation capacity. This may have introduced language bias, particularly given that flood-related outbreaks frequently occur in low- and middle-income countries where research may be published in local languages. In addition, grey literature sources, such as governmental surveillance reports and non-indexed outbreak investigations, were not systematically searched, which may have limited representation of operational public health data. Lastly, publication bias cannot be excluded, as studies with null or negative findings may be underrepresented in the literature.

Future research should focus on prospective studies with standardized outcome measures and include real-time surveillance data to better capture the dynamics of disease outbreaks. Additionally, more data are needed on the effectiveness of chemoprophylaxis and vaccines for infection prevention. Well-designed studies should be conducted to evaluate their utility in flood-related outbreak settings. Finally, research on the implications of climate change is increasingly critical. As extreme weather events become more frequent and intense, understanding how changing precipitation patterns, temperature ranges, and sea levels affect disease transmission dynamics will be essential for adaptive public health planning.

## Electronic Supplementary Material

Below is the link to the electronic supplementary material.

Supplementary Material 1

## Data Availability

All relevant data extracted for this systematic review are provided in the supplementary materials. Additional details are available from the corresponding author upon request.

## Abbreviations

aHR: Adjusted Hazard Ratio
aRR: Adjusted Rate Ratio
BOR: Bed Occupancy Rate
CFR: Case Fatality Rate
CI: Confidence Interval
DENV: 3–Dengue Virus Serotype 3
DP: Dihydroartemisinin–piperaquine
et al.: et alii (and others)
ETEC: Enterotoxigenic Escherichia coli
FY2025: Fiscal Year 2025
HAV: Hepatitis A Virus
HEV: Hepatitis E Virus
ICU: Intensive Care Unit
Inc: Incidence
IRR: Incidence Rate Ratio
JE: Japanese Encephalitis
JBI: Joanna Briggs Institute
LLIN: Long–Lasting Insecticidal Nets
LMICs: Low–and Middle–Income Countries
MAT: Microscopic Agglutination Test
MeSH: Medical Subject Headings
mg: Milligram
N/A: Not available
NIH: National Institutes of Health
OCV: Oral Cholera Vaccination
OPD: Outpatient Department
OR: Odds Ratio
P: Prevalence
PRISMA: Preferred Reporting Items for Systematic Reviews and Meta–Analyses
PROSPERO: International Prospective Register of Systematic Reviews
RDT: Rapid Diagnostic Test
ROBINS: I–Risk of Bias in Non–randomized Studies of Interventions
RR: Relative Risk
RRV: Ross River Virus
spp.: Species (plural)
STEC: Shiga Toxin–Producing Escherichia coli
U.S.: United States
USA: United States of America
US$: United States Dollar
WASH: Water, Sanitation, and Hygiene
WHO: World Health Organization
YLD: Years Lived with Disability
