# Supplementary material for: Impact of flooding events on waterborne and vector-borne infections: a systematic review
Source: BMC Infect Dis. 2026 Apr 28;26:1148. doi: 10.1186/s12879-026-13442-z (PMC13273954; doi:10.1186/s12879-026-13442-z)
Supplement: Supplementary file 1 — Supplementary Material 1 [file 12879_2026_13442_MOESM1_ESM.docx]

**Search terms:**

(“Flood*”[Title/Abstract] OR “Inundation”[Title/Abstract] OR “Natural Disasters”[MeSH Terms]

OR “Hurricane*”[Title/Abstract] OR “Cyclone*”[Title/Abstract] OR “Typhoon*”[Title/Abstract])

AND

(“Waterborne Diseases”[MeSH Terms] OR “waterborne disease*”[Title/Abstract]

OR “waterborne infection*”[Title/Abstract] OR “waterborne outbreak*”[Title/Abstract]

OR “Diarrhea, Infectious”[MeSH Terms] OR “cholera”[MeSH Terms] OR “leptospirosis”[MeSH Terms]

OR “Hepatitis A”[MeSH Terms] OR “typhoid fever”[MeSH Terms])

OR

(“Vector-Borne Diseases”[MeSH Terms] OR “vector borne disease*”[Title/Abstract]

OR “vector borne infection*”[Title/Abstract] OR “vector transmitted disease*”[Title/Abstract]

OR “dengue”[MeSH Terms] OR “malaria”[MeSH Terms]

OR “chikungunya virus”[MeSH Terms] OR “zika virus”[MeSH Terms]

OR “rickettsia”[MeSH Terms])

**Extracted data:**

1. Identifying author
2. Year of publication
3. Study design
4. Year of event(s)
5. Cause of flooding
6. Disease(s)
7. Prevalence/incidence of diseases
8. Identified Risk factors of vector-borne and waterborne disease related to flooding and their effect estimates (e.g. odds or hazard ratios) and 95% confidence intervals or alternative statistical data)
9. Identified morbidity or mortality of vector-borne or waterborne diseases related to flooding and their effect estimates (e.g. odds or hazard ratios) and 95% confidence intervals or alternative statistical data)
10. Health impact (e.g. hospitalization, icu admission) from vector borne and waterborne diseases related to flooding
11. Intervention for control outbreak

Supplementary Table S1. ****NIH Quality Assessment Tool for Observational Cohort and Cross-Sectional Studies****

| Study | Q 1 | | Q 2 | Q 3 | Q 4 | Q 5 | Q 6 | Q 7 | Q 8 | Q 9 | Q 10 | Q 11 | Q12 | Q13 | Q14 | Quality Rating |
| --- | --- | --- | --- | --- | --- | --- | --- | --- | --- | --- | --- | --- | --- | --- | --- | --- |
| Dalhat (2014) [1] | | Yes | Yes | NA | Yes | No | CD | Yes | No | NA | No | Yes | NA | NA | NA | Low |
| Ding (2014) [2] | | Yes | Yes | NA | Yes | No | Yes | Yes | No | CD | Yes | Yes | NA | NA | No | Moderate |
| Ni (2014) [3] | | Yes | Yes | NA | Yes | No | Yes | Yes | Yes | Yes | Yes | Yes | NA | NA | CD | Good |
| Wynwood (2014) [4] | | Yes | Yes | CD | Yes | No | Yes | Yes | No | No | No | Yes | NA | NA | NA | Low |
| Aumentado (2015) [5] | | Yes | Yes | NA | Yes | No | Yes | Yes | No | CD | Yes | Yes | NA | NA | NA | Moderate |
| Deng (2015) [6] | | Yes | Yes | NA | Yes | No | Yes | Yes | Yes | Yes | Yes | Yes | NA | NA | CD | Good |
| Fredrick (2015) [7] | | Yes | Yes | Yes | Yes | NR | Yes | Yes | Yes | Yes | No | Yes | NA | NA | CD | Good |
| Gertler (2015) [8] | | Yes | Yes | Yes | Yes | No | Yes | Yes | No | Yes | No | Yes | NA | NA | Yes | Moderate |
| Ito (2015) [9] | | Yes | Yes | Yes | Yes | Yes | Yes | Yes | Yes | Yes | No | Yes | NA | NA | No | Good |
| Lin (2015) [10] | | Yes | Yes | Yes | Yes | No | Yes | Yes | No | Yes | No | Yes | NA | Yes | Yes | Moderate |
| Liu (2015)[11] | | Yes | Yes | NA | Yes | No | Yes | Yes | No | Yes | No | Yes | NA | NA | No | Moderate |
| Suwanpakde (2015) [12] | | Yes | Yes | NA | Yes | No | Yes | Yes | Yes | Yes | No | Yes | NA | NA | Yes | Moderate |
| Amarnath (2016) [13] | | Yes | Yes | NA | Yes | No | Yes | CD | No | Yes | No | No | NA | NA | No | Low |
| Bloom (2016) [14] | | Yes | Yes | NA | Yes | No | Yes | Yes | Yes | Yes | No | Yes | NA | NA | No | Moderate |
| Boyce (2016) [15] | | Yes | Yes | No | Yes | No | Yes | Yes | Yes | Yes | No | Yes | NA | NA | CD | Moderate |
| Chang (2016) [16] | | Yes | Yes | NA | Yes | No | Yes | Yes | Yes | Yes | Yes | No | NA | NA | No | Moderate |
| Chirebvu (2016) [17] | | Yes | Yes | NA | No | No | Yes | Yes | Yes | Yes | CD | Yes | NA | NA | NA | Moderate |
| Gao (2016) [18] | | Yes | Yes | NA | Yes | No | Yes | Yes | No | Yes | Yes | Yes | NA | NA | Yes | Moderate |
| Gao (2016) [19] | | Yes | Yes | NA | Yes | No | Yes | Yes | No | Yes | Yes | Yes | NA | NA | Yes | Moderate |
| Liu (2016) [20] | | Yes | Yes | NA | Yes | No | Yes | Yes | Yes | Yes | Yes | Yes | NA | NA | CD | Good |
| Liu, Z (2016) [21] | | Yes | Yes | NA | Yes | No | Yes | Yes | Yes | Yes | Yes | Yes | NA | NA | CD | Good |
| Na (2016) [22] | | Yes | Yes | NA | Yes | No | Yes | Yes | No | Yes | Yes | Yes | NA | NA | NA | Moderate |
| Natuzzi (2016) [23] | | Yes | Yes | NA | Yes | No | Yes | Yes | No | Yes | Yes | Yes | NA | NA | No | Moderate |
| Pal (2016) [24] | | Yes | Yes | Yes | Yes | No | Yes | Yes | No | Yes | No | Yes | NA | NA | Yes | Moderate |
| Zhang (2016) [25] | | Yes | Yes | NA | Yes | No | Yes | Yes | No | CD | Yes | Yes | NA | NA | Yes | Moderate |
| Zhang (2016) [26] | | Yes | Yes | Yes | Yes | No | Yes | Yes | No | Yes | Yes | Yes | NA | NA | Yes | Moderate |
| Zhang (2016) [27] | | Yes | Yes | Yes | Yes | No | Yes | Yes | No | Yes | Yes | Yes | NA | NA | Yes | Moderate |
| Chan (2017) [28] | | Yes | Yes | CD | Yes | No | Yes | Yes | No | NR | No | Yes | CD | NA | NA | Low |
| Liu (2017) [29] | | Yes | Yes | NA | Yes | No | Yes | Yes | Yes | Yes | Yes | Yes | NA | NA | CD | Good |
| Xu (2017) [30] | | Yes | Yes | NA | Yes | No | Yes | Yes | No | Yes | Yes | Yes | NA | NA | Yes | Moderate |
| Zheng (2017) [31] | | Yes | Yes | Yes | Yes | No | Yes | Yes | Yes | Yes | Yes | Yes | NA | NA | No | Moderate |
| Denue (2018) [32] | | Yes | Yes | NA | Yes | No | Yes | Yes | No | CD | Yes | Yes | NA | NA | NA | Moderate |
| Elsanousi (2018) [33] | | Yes | Yes | NA | Yes | No | Yes | Yes | No | CD | Yes | Yes | NA | NA | No | Moderate |
| Hu (2018) [34] | | Yes | Yes | NA | Yes | No | Yes | Yes | Yes | Yes | Yes | Yes | NA | NA | CD | Moderate |
| Liu, Z (2018) [35] | | Yes | Yes | NA | Yes | No | Yes | Yes | Yes | Yes | Yes | Yes | NA | NA | Yes | Good |
| Mohd Radi (2018) [36] | | Yes | Yes | CD | Yes | No | Yes | Yes | Yes | Yes | Yes | Yes | NA | NA | NA | Moderate |
| Rieckmann (2018) [37] | | Yes | Yes | NA | Yes | No | Yes | Yes | Yes | Yes | Yes | Yes | NA | NA | Yes | Good |
| **Supe (2018)** [38] | | Yes | Yes | Yes | Yes | No | Yes | Yes | Yes | Yes | No | Yes | NA | NA | NA | Moderate |
| Togami (2018) [39] | | Yes | Yes | CD | Yes | No | Yes | Yes | No | NR | No | Yes | NA | NA | NA | Low |
| Adekunle (2019) [40] | | Yes | Yes | NA | NA | No | Yes | Yes | Yes | Yes | Yes | Yes | NA | NA | NA | Moderate |
| Cambaza (2019) [41] | | Yes | Yes | NA | Yes | No | Yes | Yes | CD | Yes | No | Yes | NA | NA | NA | Moderate |
| Ding (2019)[42] | | Yes | Yes | NA | Yes | No | Yes | Yes | No | No | Yes | Yes | NA | NA | No | Moderate |
| Gong (2019) [43] | | Yes | Yes | NA | Yes | No | Yes | Yes | Yes | Yes | Yes | Yes | NA | NA | Yes | Good |
| Hulland (2019) [44] | | Yes | Yes | NA | Yes | No | Yes | Yes | No | Yes | No | Yes | NA | NA | Yes | Moderate |
| Marinova-Petkova (2019) [45] | | Yes | Yes | CD | Yes | NA | Yes | Yes | Yes | NA | NA | Yes | NA | NA | NA | Moderate |
| Colston (2020) [46] | | Yes | Yes | NA | Yes | No | Yes | Yes | Yes | Yes | Yes | Yes | NA | NA | CD | Good |
| Tall (2020) [47] | | Yes | Yes | NA | Yes | No | Yes | Yes | No | Yes | Yes | Yes | NA | NA | Yes | Moderate |
| Tsai (2020) [48] | | Yes | Yes | CD | No | No | Yes | NR | Yes | CD | CD | Yes | NA | NA | NA | Low |
| Chadsuthi (2021) [49] | | Yes | Yes | NA | Yes | No | Yes | Yes | Yes | Yes | Yes | Yes | NA | NA | No | Moderate |
| Li (2021) [50] | | Yes | Yes | NA | Yes | No | Yes | Yes | Yes | Yes | Yes | Yes | NA | NA | Yes | Good |
| Silveria (2021) [51] | | Yes | Yes | NA | Yes | No | Yes | Yes | Yes | Yes | Yes | Yes | NA | NA | Yes | Good |
| Becirovic (2022) [52] | | Yes | Yes | CD | Yes | No | No | Yes | Yes | Yes | NA | Yes | NA | NA | CD | Moderate |
| Li (2022) [53] | | Yes | Yes | NA | Yes | No | Yes | Yes | CD | Yes | Yes | Yes | NA | NA | Yes | Moderate |
| Lynch (2022) [54] | | Yes | Yes | NA | Yes | No | Yes | Yes | Yes | Yes | Yes | Yes | NA | NA | Yes | Good |
| Taunton (2022) [55] | | Yes | Yes | CD | Yes | No | No | Yes | Yes | No | No | Yes | NA | NA | NA | Low |
| Balikuddembe (2023) [56] | | Yes | Yes | NA | No | No | Yes | Yes | Yes | Yes | Yes | Yes | NA | NA | NA | Moderate |
| Birhan (2023) [57] | | Yes | Yes | Yes | Yes | Yes | Yes | Yes | Yes | CD | No | Yes | NA | NA | Yes | Good |
| Bwire (2023) [58] | | Yes | Yes | NA | Yes | No | Yes | Yes | No | Yes | Yes | Yes | NA | NA | NA | Moderate |
| Sajid (2023) [59] | | Yes | Yes | NA | Yes | No | Yes | Yes | No | Yes | No | CD | NA | NA | NA | Low |
| Harris (2024) [60] | | Yes | Yes | NA | Yes | No | Yes | Yes | No | Yes | Yes | Yes | NA | NA | Yes | Moderate |
| Ifejube (2024) [61] | | Yes | NR | CD | Yes | No | Yes | Yes | Yes | Yes | NA | Yes | NA | NA | NA | Moderate |
| Jones (2024) [62] | | Yes | Yes | Yes | Yes | No | Yes | Yes | Yes | CD | Yes | Yes | NA | NA | No | Moderate |
| Luo (2023) [63] | | Yes | Yes | NA | Yes | No | Yes | Yes | Yes | Yes | Yes | Yes | NA | NA | Yes | Good |
| Nisar (2024) [64] | | Yes | Yes | NA | Yes | No | Yes | Yes | No | Yes | Yes | Yes | NA | NA | No | Moderate |

Abbreviations: CD: cannot determine; NA: not applicable; NR: not report

Q1: Was the research question or objective in this paper clearly stated?

Q2 Was the study population clearly specified and defined?

Q3: Was the participation rate of eligible persons at least 50%?

Q4: Were all the subjects selected or recruited from the same or similar populations?
Q5: Was a sample size justification, power description, or variance and effect estimates provided?

Q6: For the analyses in this paper, were the exposure(s) of interest measured prior to the outcome(s) being?
Q7: Was the timeframe sufficient so that one could reasonably expect to see an association between exposure and outcome if it existed?

Q8: For exposures that can vary in amount or level, did the study examine different levels of the exposure as related to the outcome?
Q9: Were the exposure measures (independent variables) clearly defined, valid, reliable, and implemented?
Q10: Was the exposure(s) assessed more than once over time?
Q11: Were the outcome measures (dependent variables) clearly defined, valid, reliable, and implemented ?

Q12: Were the outcome assessors blinded to the exposure status of participants?

Q13: Was loss to follow-up after baseline 20% or less?

Q14: Were key potential confounding variables measured and adjusted statistically for their impact on the relationship between exposure(s) and outcome(s)?

| Study | Q1 | Q2 | Q3 | Q4 | Q5 | Q6 | Q7 | Q8 | Quality rating |
| --- | --- | --- | --- | --- | --- | --- | --- | --- | --- |
| Yamamoto (2023) [65] | Yes | Yes | Yes | Yes | Yes | Yes | Yes | Yes | Good |
| Poulakida (2024) [66] | Yes | Yes | Yes | Yes | NA | NA | NA | Yes | Good |

Supplementary Table S2. Joanna Briggs Institute (JBI) Critical Appraisal Checklist for Case Reports

Abbreviation: NA: not applicable

Q1: Were patient’s demographic characteristics clearly described?

Q2: Was the patient’s history clearly described and presented as a timeline?

Q3: Was the current clinical condition of the patient on presentation clearly described?

Q4: Were diagnostic tests or assessment methods and the results clearly described?

Q5: Was the intervention(s) or treatment procedure(s) clearly described?

Q6: Was the post-intervention clinical condition clearly described?

Q7: Were adverse events (harms) or unanticipated events identified and described?

Q8: Does the case report provide takeaway lessons?

Supplementary Table S3. Joanna Briggs Institute (JBI) Critical Appraisal Checklist for Case Series

| Study | Q1 | Q2 | Q3 | Q4 | Q5 | Q6 | Q7 | Q8 | Q9 | Q10 | Quality rating |
| --- | --- | --- | --- | --- | --- | --- | --- | --- | --- | --- | --- |
| Zaghi (2024) [67] | Yes | Yes | Yes | NA | Yes | Yes | Yes | Yes | Yes | NA | Good |

Abbreviation: NA: not applicable

Q1: Were patient’s demographic characteristics clearly described?

Q2: Was the patient’s history clearly described and presented as a timeline?

Q3: Was the current clinical condition of the patient on presentation clearly described?

Q4: Were diagnostic tests or assessment methods and the results clearly described?

Q5: Was the intervention(s) or treatment procedure(s) clearly described?

Q6: Was the post-intervention clinical condition clearly described?

Q7: Were adverse events (harms) or unanticipated events identified and described?

Q8: Does the case report provide takeaway lessons?

**Supplementary Figure S1*.*Risk of Bias Assessment Using ROBINS-I Tool for Non-Randomized Studies of Interventions**


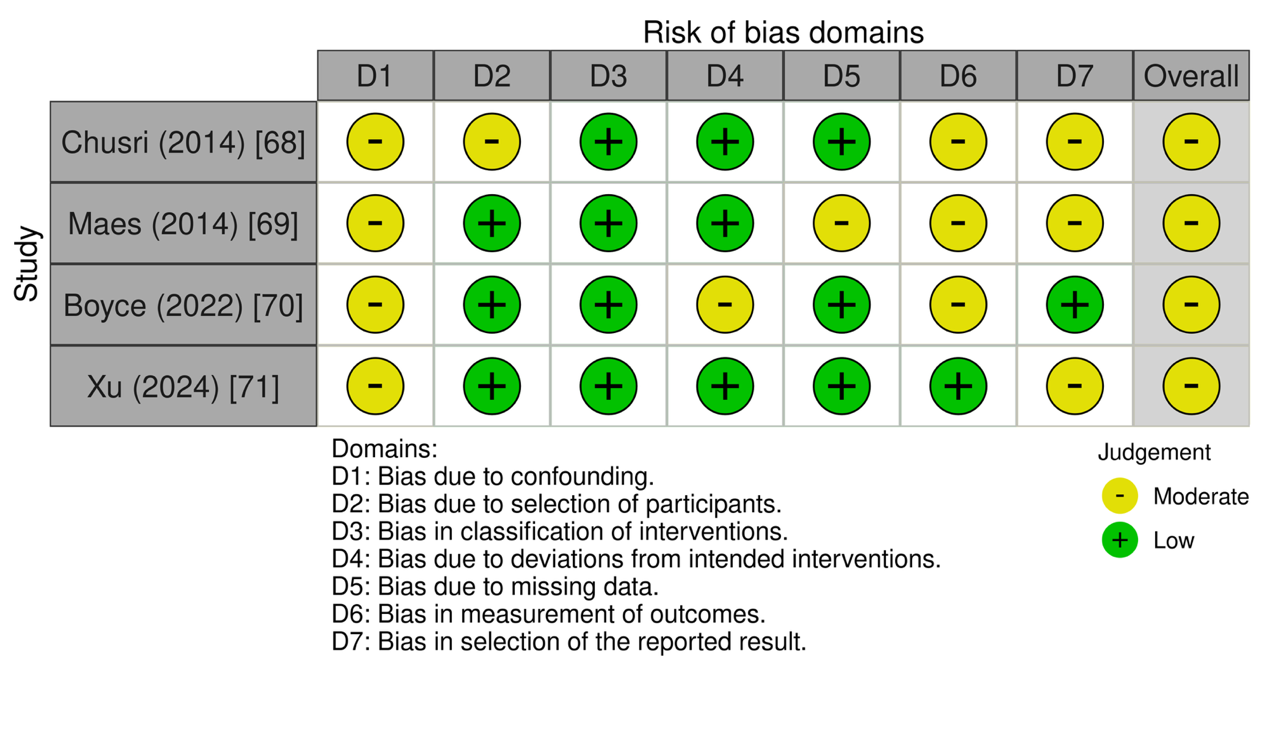


**Supplementary Table S4. Summary of Trials on Leptospirosis Prevention**

| **Study** | **Study Design** | **Flood Description**  **(Cause/Year)** | **Country** | **Population** | **Sample Size** | **Intervention** | **Comparator** | **Outcome** |
| --- | --- | --- | --- | --- | --- | --- | --- | --- |
| Chusri (2014) [68] | Prospective, non-randomized controlled study | Unknown/2010 | Thailand | Adults ≥18 exposed to floodwater | 600 (intervention); 41 (comparator) | Doxycycline 200 mg single dose; start 1–3 d post-flood | Non-prophylaxis group | ↑ Protective efficacy: 76.8% |
| **Supe (2018) [38]** | Descriptive observational study | Unknown/2017 | India | Flood-exposed urban population | 6.7 million (screened); 166,715 (prophylaxis) | Doxycycline 200 mg (single / 3 d / weekly×6 wk); azithromycin for children/pregnant; SMS/media support | Non-prophylaxis year (2005) | (-) Confirmed cases: 59 (2017) vs 432 (2005) |

Abbreviations: ↑: Statistically significant increase; (-): decrease; d: day(s); wk: week(s); mg: milligram

**Supplementary Table S5. Summary of Trials on Malaria Prevention**

| **Study(year)** | **Study Design** | **Flood Description**  **(Cause/Year)** | **Country** | **Population** | **Sample Size** | **Intervention** | **Comparator** | **Outcome** |
| --- | --- | --- | --- | --- | --- | --- | --- | --- |
| Maes (2014) [69] | Observational | Unknown/ 2006-2007 | Kenya | All inpatient and outpatient malaria cases during 1996 -1998 and 2005 -2007 | N/A | Timely IRS, LLINs, larviciding (2005-2007) | **Delay** IRS, LLINs, larviciding  **(1997-1998)** | **Delayed intervention : 54/1,000 weekly inc, 28/10,000 child mortality;** **Timely intervention: <0.5/1,000 weekly inc, no child mortality** |
| Boyce (2022) [70] | Quasi-experimental observational | Unknown/2020 | Uganda | Children aged ≤12 years | N/A | Monthly DP chemoprevention for 3 consecutive months post-flood (Intervention village) | No intervention  (control village) | ↓Inc (per 1,000 person-weeks): 9.4 vs 20.2 cases; aRR= 0.47 |

Abbreviations: ↓: Statistically significant decrease; aRR: adjust rate ratio; DP: Dihydroartemisinin-Piperaquine; Inc: incidence; IRS: Indoor residual spraying; LLINs: long-lasting insecticidal nets; N/A: not availabl
